# Critical Analysis of Protocols for Good Veterinary Practices in Monitoring, Prevention and Treatment of Ketosis in Dairy Cows

**DOI:** 10.3390/vetsci12101019

**Published:** 2025-10-21

**Authors:** Elena Stancheva, Toncho Penev

**Affiliations:** Department of Ecology and Animal Hygiene, Trakia University, 6000 Stara Zagora, Bulgaria; tonchopenev@abv.bg

**Keywords:** clinical ketosis, subclinical ketosis, monitoring, good practices

## Abstract

Ketosis is one of the most common metabolic disorders in high-yielding dairy cows in early lactation. It has a negative impact on milk yield, reproduction, and general health of the animals. The present review aims to systematize and critically analyze current scientific data on the monitoring, diagnosis, prevention, and treatment of subclinical and clinical ketosis, with the aim of developing an applicable protocol for good veterinary medical practices (GMP). Based on the comparative analysis method of data from the literature and clinical practice, β-hydroxybutyrate (BHBA) in blood is confirmed as the gold standard for diagnosis with specificity and sensitivity above 90%. Indicators such as fat/protein (F/P) > 1.4 and NEFA > 0.4 mmol/L, as well as reduced citrate content in milk, have been evaluated as useful screening tools, although with lower diagnostic value. Despite the advantages of some indirect methods (such as F/P), critical analysis shows that they are strongly influenced by physiological status, lactation stage, and diet and cannot replace direct blood tests. Preventive approaches emphasize the importance of stable nutrition in the pre- and post-calving period, restriction of ketogenic feeds, and use of oral glucose precursors. Literature analysis shows that propylene glycol is effective, but with prolonged use it can reduce appetite. Combined antiketotic products have also been introduced, providing not only energy support but also liver protection. Particular attention has been paid to monensin (applied in the commercial product “Kexxtone”)—a polyether antibiotic with sustained release, which reduces the incidence of ketosis by over 50% and increases milk yield in the first weeks after calving. However, its high cost, antibiotic nature, and need for veterinary supervision limit its universal use. The treatment protocol is differentiated according to the clinical type: glucose I. V. and propylene glycol in type I ketosis and avoidance of glucocorticoids in suspected type II (hepatic steatosis). In the critical analysis It is noted that improper use of glucocorticoids can lead to a worsening of the condition. A structured protocol for DVMP (Dairy Veterinary Medical Practice) is proposed, which includes targeted metabolic monitoring (NEFA, BHBA, F/P); proven preventive regimens (Kexxtone, propylene glycol, balanced rations), and staged prevention and treatment according to the form of ketosis. The adaptation of good practices to the scale of the farm and the level of knowledge and skills of the staff for their correct application remains a challenge. Providing training, a standardized control log, and access to field diagnostic tools is key to the success of any protocol.

## Simple Summary

Ketosis is a common metabolic disorder in dairy cows, causing productivity losses and economic impact. This review critically analyzes all current monitoring, prevention, and treatment methods, evaluating their effectiveness and practical use. A structured protocol for good veterinary medical practices (GMP) is proposed, helping veterinarians and farmers control the disease efficiently and support sustainable dairy farm management.

## 1. Introduction

Ketosis is a metabolic condition predominantly impacting high-yielding dairy cows. It may arise during the transition phase, the dry period, at calving, or during early lactation [1]. The highest incidence of ketosis occurs during the first 2 to 3 weeks of lactation [2]. The disorder is characterized by high concentrations of circulating ketone bodies (acetone, acetoacetate, and *β*-hydroxybutyrate) even in the absence of clinical signs of ketosis [3]. This condition is a major determinant of cow health and productivity throughout lactation and causes economic losses due to reduced milk production [4], the occurrence of diseases such as displaced abomasum [5], and impaired reproductive status [6]. Subclinical ketosis (SCK) is associated with negative energy balance in high-producing dairy cows. After calving, milk yield increases rapidly, while dry matter intake remains limited, forcing cows to mobilize body fat reserves to meet energy demands. The mobilized fatty acids are metabolized in the liver and converted into ketone bodies [7]. SCK occurs when there is an elevated concentration of ketone bodies, particularly BHBA, in the blood. Although the clinical signs may be mild or even absent, this condition can still lead to considerable economic and reproductive consequences [8,9].

Given the growing importance of early detection and prevention of metabolic disorders in dairy herds, ketosis remains a key challenge in herd health management. The present review aims to provide a critical analysis of the established protocols for monitoring, prevention, and treatment of ketosis in dairy cows, summarizing practical approaches and their effectiveness as part of good veterinary practice (GVP).

## 2. Purpose and Objectives

The main objective of this paper is to perform a critical analysis of the protocols for good veterinary practices in monitoring, prevention, and treatment of ketosis in dairy cows.

To achieve our goal, we set the following tasks:

(1) to study the good veterinary practices established as such for monitoring and controlling ketosis in the scientific literature;
(2) to conduct a critical analysis of the effectiveness of such established practices;
(3) to offer future perspectives for the development of good practices for ketosis control.

## 3. Materials and Methods

A structured descriptive review was performed to compile and analyze published evidence and practical data on ketosis management in dairy cows.

Scientific and popular science articles and websites were used as material for this article.

The following methods were used to compile this article:

(1) Sources and databases used for search:

The literature was selected by searching reputable scientific databases, including:

- ScienceDirect (Elsevier)
- PubMed (NCBI)
- SpringerLink
- Google Scholar (if you need access to extended texts)
- Veterinary conferences and proceedings: World Buiatrics Congress, Vet. Clin. North Am., J. Dairy Sci.

(2) Method of defining a thematic framework

The selection of literature was carried out purposefully according to main sub-topics related to ketosis in dairy cows:

- Metabolism and etiology of ketosis (type I, II, silage ketosis).
- Diagnostic methods based on the detection of BHBA, NEFA, citrate, fat/protein (F/P) ratio in milk.
- Therapeutic approaches based on the application of proven products such as propylene glycol, glucose, glucocorticoids, and monensin.
- Prevention and monitoring at the herd/farm level.

Objective: To build a complete scientific context that covers the physiology, diagnosis, treatment, and prevention of clinical and subclinical ketosis.

(3) Selection process:

- Keywords and search—used combinations such as:
- “subclinical ketosis in dairy cows”, “BHBA monitoring”, “monensin prevention dairy cattle”, “milk fat to protein ratio—ketosis”
- Review of titles and abstracts—primary selection by keywords
- Analysis and evaluation of the full text
- Data extraction and citation in standard format (APA)

(4) Survey of Veterinary Practitioners and Companies

A survey method among practicing veterinarians and companies selling veterinary medicinal products on the schemes, protocols, and products used for monitoring and controlling ketosis in cows. The study involved surveying practicing veterinarians and representatives of companies supplying veterinary medicinal products regarding the schemes, protocols, and products used for monitoring and controlling ketosis in cows. The collected information was systematized and used to conduct a systematic review of practical approaches and their effectiveness in the context of published data from specialized veterinary sources.

**Figure 1:**
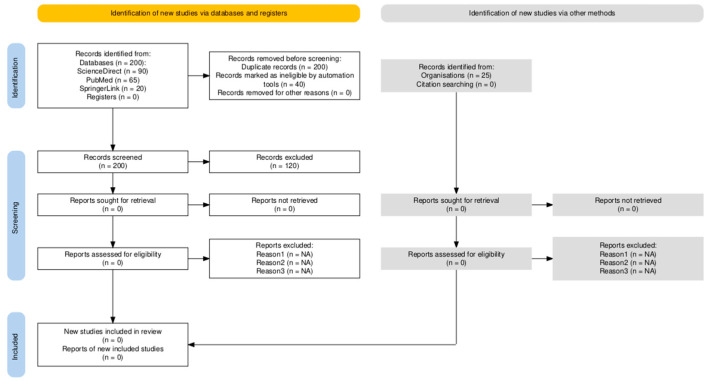
The process of identification, screening, and inclusion of publications is presented in the PRISMA 2020 flow diagram.

(5) Categorization and integration:

After selection, the literary sources were distributed into thematic blocks:

- Etiology and pathogenesis
- Diagnostic methods and values
- Treatment strategies
- Prevention/feeding/monitoring

(6) The criteria for inclusion of the literature are described in Table 1. The process of identifying, screening and including publications is shown in Figure 1.

This allowed for the creation of tables, critical analysis, and summaries with a clear logical transition and scientific value.

The selected literature is the result of a rigorous, multi-component process of search, selection, and critical evaluation, aimed at providing up-to-date, reliable, and applicable knowledge about ketosis in dairy cows, with an emphasis on practical prevention and treatment in modern cattle breeding.

As part of the study, a systematic literature review was conducted in accordance with the PRISMA guidelines (Preferred Reporting Items for Systematic Reviews). The methodological approach included defining inclusion and exclusion criteria for sources, systematically searching for relevant publications in scientific databases, and performing a critical evaluation of their content, summarized in Table 2.

The review was prepared and reported in accordance with the PRISMA 2020 guidelines.

## 4. Monitoring

Several classification schemes for ketosis have been reported in the scientific literature, and two different approaches have been most commonly used by different investigators. The first categorization is based primarily on blood BHBA concentrations and the absence or presence of clinical signs of disease. Accordingly, ketosis is classified into two forms, including subclinical ketosis (SCK) and clinical ketosis (CK). Subclinical ketosis (SCK) is defined as an increase in ketone bodies in the blood, urine, or milk in the absence of obvious clinical signs of disease. The cow maintains appetite and does not have a decrease in dry matter intake (DMI). Serum BHBA concentrations between 1200 and 1400 µmol/L are commonly used to diagnose subclinical ketosis (SCK) [10]. Clinical ketosis (CK) is characterized by hyperketonemia, hypoglycemia, and the presence of clinical signs, including decreased appetite, weight loss, and decreased milk production [11]. Clinical ketosis is usually recognized by elevated blood BHBA concentrations, ranging from 2600 to 3000 µmol/L [2,12]. A summary of blood BHBA concentrations and their significance is presented in Table 3.

The second classification scheme categorizes ketosis based on the etiology and timing of hyperketonemia into the following three main types: ketosis type I, ketosis type II, and silage ketosis arising from butyric acid [12,13,14].

Type I, or primary, ketosis is the classic form of ketosis that occurs between 3 and 6 weeks after calving, when energy loss through milk reaches its peak [13]. It is designated as type I ketosis because it is similar to type I diabetes mellitus in humans. Cows with type I ketosis experience hypoinsulinemia at the time of diagnosis of hyperketonemia due to chronic hypoglycemia resulting from a lack of glucose precursors for milk production. In this case, glucose precursors are absorbed from the diet (mainly propionate) or from muscle proteins in the form of amino acids (AA, i.e., gluconeogenesis). However, the capacity of the gluconeogenic process is limited due to host protection of muscle proteins. On the other hand, lipolysis and ketogenesis are enhanced, with fatty acids and ketone bodies being used as a source to preserve glucose and meet energy needs [13]. In this type of ketosis (characterized by increased fat content and decreased protein content in milk), it is necessary to take targeted actions to increase glucose precursors in the ration in order to correct the negative energy balance. The most effective solutions include the application of propionates—mainly in the form of propylene glycol or sodium propionate.

Both approaches have specific advantages and limitations. Oral individual administration of products (as boluses or via dispensers) provides guaranteed and precise dosing but is associated with greater physical effort, the need to restrain the animals, and the risk of stress. On the other hand, the addition of glucose precursors directly to the ration (e.g., via TMR) significantly facilitates administration and reduces labor but does not allow precise control over the amount taken by each animal, which may compromise the effectiveness of the therapy, especially in high-risk or already affected cows.

Type II, or secondary, ketosis usually occurs immediately after birth and is associated with other conditions such as fatty liver [13]. This form of ketosis is called type II ketosis based on its human metabolic counterpart, type II diabetes mellitus. Cows with type II ketosis have high concentrations of both insulin and glucose in the blood and are diagnosed with hyperketonemia [13]. Insulin resistance may also exist during type II ketosis [12]. Obesity and overfeeding during the dry period are critical for the development of this type of ketosis. Mobilization of body fat from adipose tissue and accumulation of triglycerides (TG) may occur before or during birth [12]. Excessive accumulation of TG in the liver not only impairs gluconeogenesis but also suppresses the immune functions of hepatocytes. The actions in this type of ketosis are mainly associated with feeding management during late lactation and throughout the dry period. It is unacceptable to allow cows to become obese with a BCS above 3.5–4. Monitoring was performed by assessing BCS during the dry period, as a higher BCS at dry-off is associated with an increased risk of this type of ketosis, while a rapid drop in BCS can serve as an indicator for early detection.

Ketosis associated with the consumption of silage rich in butyric acid is the third type of ketosis according to the second categorization scheme. This type of ketosis is due to the intake of feed with a high content of ketogenic precursors (i.e., butyric acid) [7]. The reasons for the accumulation of butyric acid in silage are related, for example, to the preparation of silage from hay that contains low water-soluble carbohydrates or silage that is prepared with too high a water content, which favors the growth of bacteria of the genus *Clostridium* sp. [12]. Some carbohydrates in silage are fermented to butyric acid, rather than the preferred lactic acid. Cows develop silage ketosis from butyric acid when they ingest large amounts of silage that has undergone clostridial fermentation. However, whether or not a cow will develop butyric acid ketosis as a result of feed intake also depends on the amount of silage consumed and the presence of other risk factors (e.g., early lactation, rumen acidosis, high milk production, low ration energy, and high ration protein) for ketosis [12]. Analysis of silage quality may represent a potential means for monitoring this type of ketosis. A comparative description of the types of ketosis is presented in Table 4.

Cases of hyperketonemia occurring near the time of peak milk production (usually around 4–6 weeks postpartum, but sometimes as early as 2 weeks postpartum) are associated with malnourished cattle experiencing a metabolic deficiency of gluconeogenic precursors rather than excessive fat mobilization. This may be due to the fact that high-producing dairy cows expend a large amount of energy in milk production or because their dietary energy requirements are not being met, i.e., the ration does not provide for their needs [15].

Associated signs are:

In stage I of the illness, cows tend to prefer hay and grass over concentrated feeds and silage. They may consume sand and gravel, exhibit hesitation in movement, display an unsteady walk, and keep their heads elevated while resting.

In stage II of the disease, cows eat significantly less and are “dull” and apathetic. Milk production decreases, and animals lose weight. The milk has a high fat content (sometimes up to 5%) and a small amount of protein. The breath expelled carries a scent reminiscent of acetone and rotting fruit. Moreover, the cow dung appears hard, dry, and coated in mucus. The cows keep their heads lowered and eyes closed as if they are dozing off.

In stage III of the illness, cows frequently advance aimlessly, exhibiting convulsions along with excessive drooling. These animals display heightened sensitivity to sound and touch, producing sounds that resemble drinking (they place their snouts in the water trough and chew loudly). The blood analysis reveals significantly reduced concentrations of circulating lipids, specifically triglycerides and non-esterified fatty acids (NEFA), coupled with elevated levels of BHBA in the cows’ bloodstream [16]. From what has been said so far, it is clear that the clinical signs of ketosis are unclear and often with an uncertain result. It can be confidently stated that in order to diagnose ketosis only by clinical signs, an individual approach to each animal is necessary. This can only be applied in small family farms and by the owners or keepers of the animals. In industrial dairy farming, this individual approach to diagnosis based only on clinical signs and behavior is impossible. Such an individual approach can only be applied by monitoring the amount of milk produced, information about which is provided by the herd management software. The next step in the diagnosis process is to isolate the animal and conduct paraclinical tests—blood tests and, less commonly, a milk test for ketone body content. Table 5 presents preventive strategies for ketosis control in dairy cows.

Notes:

- Preventive measures must be combined with good transition-cow management and nutritional consistency.
- Monensin usage depends on regional regulatory approval; veterinary oversight is mandatory.
- Practical application is strongest when prevention is integrated into herd-level metabolic monitoring.

Preventive Measures and Critical Analysis Table 5 presents preventive approaches that integrate nutritional, metabolic, and management strategies. 

Transition-Cow Management

Transition period management is considered the cornerstone of ketosis prevention. Recent reviews [17,18] emphasize that controlling body condition score (BCS, 3.0–3.5 at calving) and maintaining high dry matter intake (DMI) have the strongest level of evidence. Our observations fully support this conclusion: farms with optimized BCS and balanced transition diets show a lower prevalence of subclinical ketosis.

Key conclusion: Proper transition-cow management consistently reduces the risk of subclinical ketosis.

Monensin Usage

Monensin (CRC) can have a preventive effect in high-risk cows, but its effectiveness varies between herds and regions due to differences in diet, management, and local conditions [18,19,20]. Its use must comply with regional regulations and always involve veterinary supervision.

Key conclusion: Monensin is a useful tool under specific conditions but should not be considered a universal solution.

Short-Term Nutritional Supplements

Short-term supplements such as propylene glycol, glycerin, and calcium propionate improve glycemia and BHBA levels. However, there is insufficient long-term evidence regarding their impact on reproductive performance. Our results indicate that optimal outcomes are achieved when nutritional management is combined with targeted supplementation rather than relying on a single strategy.

Key conclusion: Combined nutritional and targeted supplementation strategies are more effective than unilateral approaches.

### 4.1. Fat/Protein Ratio (F/P) in Milk as a Practical Indicator of the Presence of Ketosis

In recent years, increasing attention has been paid to the fat/protein (F/P) ratio of milk, especially in the early stages of lactation. The main reason is the possibility of using it as an important criterion for energy balance in the assessment of cow nutrition.

Fat is produced in the mammary gland by combining glycerol with fatty acids that are synthesized from acetic acid within the same gland (known as de novo synthesis). Some of these fatty acids originate from feed or are liberated from adipose tissue in the form of non-esterified fatty acids (NEFA). It is well recognized that fat reserves are often mobilized during the perinatal phase in dairy cows. When there is an energy deficit relative to their daily requirements, and if pregnant cows experience excessive weight gain leading to reduced appetite, this results in their energy needs not being adequately met. Consequently, these cows become weaker and release fatty acids into their bloodstream. A substantial portion of these fatty acids makes its way into the mammary gland, thus increasing both fat content and the proportion of saturated fats in milk [16].

The protein level found in cow’s milk relies on both the dietary intake affecting rumen microorganisms’ energy supply and the overall protein consumed by the animals. However, if there is not enough energy supplied to facilitate microbial synthesis or if there is an overabundance of protein manifested as ammonia within the rumen, it will be converted via body mechanisms into urea for excretion (such as through milk) [16].

For a healthy cow receiving a nutritionally balanced diet, the fat/protein (F/P) ratio should fall between 1.1 and 1.3:1. An F/P ratio above 1.4:1 raises concerns regarding potential subclinical ketosis (SCK), while clinical ketosis may occur when this ratio surpasses 1.7:1—particularly when accompanied by low protein levels and high fat content in milk [21].

Cows producing >5% milk fat within 41 days post-calving alongside less than 2.90% milk protein require special monitoring. Should more than 10% of cows exhibit abnormal milk composition—characterized by elevated fat but insufficient protein—during lactation days 7 to 60, this could indicate an energy imbalance across the herd along with increased risk for subclinical ketosis. In such circumstances, conducting metabolic screenings such as blood β-hydroxybutyrate measurements would be prudent alongside necessary adjustments to nutrition and management practices.

The F/P ratio serves as a gauge for evaluating a cow’s energy balance during early lactation [22]. SCK can be indicated by elevated milk fat levels and increased BHBA concentrations, which can be measured in blood or milk using specific ketone test kits to provide an indirect assessment of the cow’s metabolic status.

Eicher [23] advocates for utilizing both F/P ratios and daily yield metrics to identify instances of subclinical ketosis effectively. He further notes that an F/P ratio ≥1.5 among cows yielding between 33 and 50 kg/day can also serve as a reliable indicator for detecting SCK.

Methods for diagnosing ketosis

Methods for determining ketosis are direct and indirect.

Direct methods include:

Determining the level of ketone bodies in the blood

This technique is primarily employed by veterinarians for the diagnosis of clinical ketosis (CK). It is an intrusive and costly approach, making it impractical for routine use across all animals. Generally, only cows displaying significant clinical symptoms undergo blood tests. Analyzing serum levels of NEFA, BHBA, and glucose proves to be highly effective in diagnosing ketosis in dairy cows. Measuring blood sugar poses no major issues; however, a significant limitation lies in the specificity required, necessitating access to a specialized laboratory for assessing the other two parameters. Additionally, it is feasible to gauge ketone body concentrations in the blood of dairy cows using glucometers designed for human use. The glucometer detects electrochemical alterations occurring on the test strip upon blood application, which enables its functionality for both humans and cattle. The strip used contains 3-hydroxybutyrate dehydrogenase—an enzyme that carries out the oxidation of BHBA when exposed to an alternating current (AC). This in turn reduces nicotinamide adenine dinucleotide (NAD+) to NADH, and then NADH is re-oxidized to NAD+. The electric current generated during this transformation is directly proportional to the concentration of βHBA in the blood. It seems very easy to measure. However, some experience is required to take blood from cows. According to Polish law, blood can only be taken by a veterinarian. The first report using an electronic human BHBA meter (MediSense Precision, Abbott, Abingdon, UK) for dairy cows described a high correlation (r^2^ = 0.99) with BHBA concentrations determined spectrophotometrically (gold standard), and the test was considered suitable for the detection of subclinical ketosis (SCK) in dairy cows [24].

#### Determination of Ketone Bodies in Milk or Urine

Several tests for the diagnosis of SCK in cows are commercially available (sticks, powders, and tablets). They are designed to detect acetoacetate and, to a lesser extent, acetone in urine (e.g., Ketostix strip, Bayer, Leverkusen, Germany) or BHBA in milk (e.g., Ketolac, Biolab, München, Germany) based on the degree of color change. The tests can be used semi-quantitatively, as the color change is more intense in the presence of higher levels of ketone bodies [25]. However, these color-based methods have their own subjective nature due to the color perception of the person performing the test, as well as the level and characteristics of the light under which the result is read.

Based on the studies conducted, we systematized the data and prepared a protocol reflecting the comparison of advantages and disadvantages when choosing an indirect method for detecting SCK in dairy cows.

Indirect determination of ketosis includes:

Clinical signs (loss of appetite, preference for roughage over concentrates, and acetone odor in breath and urine), change in milk composition, increase in diseases (mastitis, metritis, and displaced abomasum), and reproductive disorders.

High plasma concentrations of BHBA and non-esterified fatty acids (NEFA), high levels of milk fat and citrate (the latter being considered an early indicator), and lower concentrations of milk protein and lactose are characteristics of ketosis. The levels of BHBA, NEFA, and citrate are higher in the blood of cows suffering from ketosis than in other cows [26].

According to current knowledge, the basal ration for dry cows should be limited to 75–80% of the nutritional standards, and the amount of straw should be increased to prevent type II ketosis. This will lead to a slower release of fat reserves after calving [27]. Detailed recommendations for preventing ketosis are to keep cows in good condition (with a BCS between 3.0 and 3.5 at calving), feed cows the same feed 3 weeks before calving and after calving, add 0.5–3 kg of concentrate feed to the ration immediately before calving, carefully balance the feed ration (correct protein-energy ratio), and avoid ketogenic feeds (silage contains butyric acid and feeds with a high concentration of easily digestible carbohydrates). Table 6 presents the diagnostic methods for ketosis in dairy cows.

Notes:

- Combined diagnostic approach recommended:
  1. Screening—by milk F/P ratio or milk BHBA;
  2. Confirmation—by blood BHBA measurement;
  3. Risk monitoring—by prepartum NEFA sampling.

The diagnostic methods summarized in Table 6 reflect the evolution of approaches for the early detection of ketosis, ranging from classical laboratory analyses to modern rapid tests. According to [17], blood BHBA concentration remains the most reliable biochemical marker and the “gold standard” for confirming the diagnosis. Our review aligns with this conclusion, adopting a threshold of ≥1.2 mmol/L as an optimal compromise between sensitivity and specificity. Other authors, such as [1,18] suggest broader ranges (0.85–1.4 mmol/L) depending on breed and the analytical device used, highlighting the need for context-specific threshold adjustments.

Regarding milk parameters, several studies [19,28] confirm that a fat-to-protein ratio (F/P) > 1.4 is a useful indicator for herd-level screening, although its individual diagnostic value is limited—particularly during dietary changes or different lactation stages. Our position aligns with [17] in that a combined algorithm—milk F/P or milk BHBA → confirmation with blood BHBA → supplementary NEFA profile—provides the greatest practical value.

According to [18,20], NEFA should be used primarily prepartum as a prognostic marker rather than for routine diagnosis, a stance we also support. In summary, we consider a multi-indicator approach—a combination of BHBA, F/P, and NEFA—to be the most effective and economically justified strategy for diagnosing ketosis in dairy cows.

## 5. Ketosis Treatment

The individual or group administration of supplements can help prevent ketosis in dairy cows by improving energy balance and reducing the risk of subclinical and clinical manifestations. Oral glucose precursors, such as glycerin, propylene glycol, and calcium or sodium proprionate, should be given to herds particularly susceptible to ketosis for a duration of 7–10 days prior to calving and continuing up to 2 weeks afterward. Incorporating these substances into the diets of cows helps in preventing excessive depletion of fat stores, lowering levels of free fatty acids, and boosting triglyceride along with insulin concentrations. One significant benefit of administering glucose precursors is their ability to mitigate the likelihood of developing fatty liver syndrome. Generally speaking, it is advisable to administer multiple types together (for instance, glycerin, propylene glycol, calcium, or sodium proprionate), as different glucose precursors elevate blood glucose levels at varying rates. The recommended daily dosages are between 250 and 300 g/day approximately two weeks before calving, increasing to 300–350 g/day after calving until around 100 days post-lactation; these compounds can be mixed into either the feed or during feed preparation. For preventive measures after calving, a tailored antiketotic solution may be formulated using the following ingredients: 230 mL propylene glycol, 450 g calcium proprionate, 50 g rumen-protected choline, 25 g rumen-protected methionine, 50 g yeast, and 170 g KCl dissolved in roughly 5 L of warm water (40–45 °C) [28].

They should be administered orally as an infusion in severe symptoms of ketosis (anorexia and lack of energy). Feed glycerin is a sweet natural product produced from rapeseed. It provides the necessary energy to cows after calving and improves the palatability of feed, increasing its intake and preventing ketosis. Several products—oral glucose precursors—are available on the market.

Glucose administration is also a mainstay of treatment for subclinical and clinical ketosis (CK). Its undoubted advantage is that its blood concentration increases very rapidly when administered intravenously. It is recommended that glucose be administered together with vitamin B1 or multivitamins. Glucocorticoids are used as a second-line treatment for ketosis. Their advantage is the ability to increase blood sugar levels rapidly and for long hours (48–72 h after administration). After administration of glucocorticoids, the general health condition improves and the concentration of BHBA decreases. However, at the same time, there is a temporary decrease in daily milk yield [28]. It should be emphasized that this drug is not indicated in case of type II ketosis, when the ketosis-hepatic steatosis complex is well developed.

The medication that features monensin as its active ingredient is also utilized in the prevention of ketosis. Monensin, a polyether antibiotic derived from Streptomyces cinnamonensis [29], lowers the rate of ketosis by influencing microbial communities within the rumen when given to cattle. It inhibits the intracellular transport of specific ions and suppresses bacteria that produce lactate. While treatment with monensin effectively eradicates Gram-positive bacteria, it does not affect Gram-negative strains. This alteration in rumen flora instigates metabolic shifts in livestock, enhancing energy and nitrogen assimilation from their feed. As a result, there is a notable improvement in nutrient uptake while simultaneously diminishing the chances of digestive issues such as rumen bloat [16].

Kexxtone is a veterinary medication that features monensin as its active ingredient and comes in a sustained-release formulation (the bolus is administered orally to the animals, allowing it to enter the rumen). The term “sustained release” indicates that the monensin gradually disperses from the bolus. A single intraruminal bolus is provided to dairy cows or heifers approximately 3–4 weeks prior to their anticipated calving date. The rates of ketosis were observed at 11.5% for those receiving Kexxtone compared to 25.6% in the placebo group. Additionally, treated cows showed a tendency toward significantly greater milk production within the first 14 days of lactation [30]. The drug “Kexxtone” is a plastic capsule with a small hole on one side and “wings” on the other. The “wings” provide support for the keystone in the upper layers of the contents of the rumen. The latter, through the hole, comes into contact with the active ingredient of “kexxton”. Before the capsule is administered, the animal is held so that the head and neck are in an upright, extended position. The capsules are folded into the “wings” and then placed with the free end in the bolus applicator. The applicator is introduced into the oral cavity behind the back of the tongue, not allowing it to touch the molars. Disintegration occurs after gradual dissolution of the monensin tablets, with one of the “wings” breaking off, allowing the cow to push the plastic tube along with the burp [31].

One application of “Kexxtone” provides protection for 95 days during the period when cows are most prone to ketosis. Kexxtone is applied 3–4 weeks before the planned calving to provide protection for 10 weeks after calving [31].

The Kexxtone product is presented in tabular form. Monensin (in the form of Kexxtone) is a proven, long-acting prophylactic agent against ketosis, with a single administration and long-term release. It is suitable for large herds, especially for cows at high risk of metabolic diseases after calving. However, it requires careful handling and veterinary supervision. Table 7 presents therapeutic options for the treatment of ketosis in dairy cows.

Notes:

- Propylene glycol remains the gold-standard therapy for subclinical ketosis.
- Combining glucose infusion with glucocorticoids may be beneficial in severe clinical cases but should be administered under veterinary control.
- Avoid repeated corticosteroid use in lactating cows due to risk of immunosuppression.
- Integration of therapy with herd-level metabolic monitoring ensures sustained recovery and reduces relapse rate.

Critical Analysis Table 7 presents the main therapeutic agents and treatment protocols for ketosis in dairy cows. Comparison with recent reviews [17,18] shows consensus that propylene glycol remains the first-choice treatment for subclinical and mild clinical forms due to its availability, proven efficacy, and safety. Our own field observations also support this conclusion.

Key conclusion: Propylene glycol is the first-choice treatment for subclinical and mild clinical ketosis.

Intravenous glucose provides a rapid but short-lived effect and should be combined with oral glucogenic precursors to maintain the response—an approach also supported by [1]. Glucocorticoids (dexamethasone and isoflupredone acetate) can stimulate gluconeogenesis, but several authors [18,20] caution about the risk of immunosuppression and induced parturition. Our position is that their use should be limited to severe cases and always under veterinary supervision.

Recommendations:

Use glucose and glucocorticoids only in severe clinical cases.

Always administer under veterinary supervision.

Avoid repeated corticosteroid use in lactating cows due to immunosuppression risk.

Key conclusion: Glucose infusion and glucocorticoids can be effective in severe cases but must be carefully managed.

The lack of large randomized trials with long-term outcomes necessitates caution when comparing therapeutic protocols. Therefore, we support the recommendation of [17] for an individualized, staged approach, in which the response is monitored via BHBA levels and appetite, and therapy is dynamically adjusted.

Key conclusion: Individualized, staged therapy guided by herd-level monitoring optimizes outcomes and minimizes risk of recurrence.

## 6. Discussion

The literature review identified several risk factors/moments that would aid in the early detection and monitoring of ketosis in dairy cows.

The introduction of a good veterinary practice protocol regarding the monitoring, prevention, and treatment of the metabolic disorder ketosis is essential.

The main critical points that we need to address from the monitoring side include:

Protocol for monitoring, prevention, and treatment of ketosis in dairy cows

1. Purpose of the Protocol:
  - On-time identification of animals at risk
  - Early diagnosis of subclinical ketosis
  - Prevention of clinical complications
  - Optimizing productivity and reproduction
2. Monitoring: Indicators, limit values, and measurement methods;
3. Preventive strategies: best nutritional and management strategies;
4. Prevention and treatment by stage of ketosis.
  - Table 8 presents the integrated GVP protocol for monitoring, prevention, and treatment of ketosis in dairy cows.

Notes:

- BHBA ≥ 1.2 mmol/L indicates subclinical ketosis (SCK); ≥3.0 mmol/L indicates clinical ketosis (CK).
- Integration of monitoring, prevention, and treatment ensures effective herd-level control.
- Protocols should be adjusted to local regulations and farm management conditions.
- This GVP model aligns with the Dairy Veterinary Medical Practice (DVMP) framework and recent reviews [17,28].

Critical Analysis: Table 8 presents a synthesized integrated protocol based on the Good Veterinary Practice (GVP) concept, adapted to the framework of Dairy Veterinary Medical Practice (DVMP). This approach combines diagnostic thresholds, preventive strategies, and therapeutic interventions into a single, practical scheme. Comparison with published reviews [17,19,28] shows a high degree of alignment—particularly regarding the prioritization of transition period management and combined monitoring using BHBA and F/P.

Our position is that the effectiveness of such an integrated protocol depends on its flexibility and adaptation to the specific farm context. Some authors [18,20] emphasize the need for more detailed economic evaluation of implementing such schemes, especially under varying resource availability. We agree with this view and consider the proposed framework as a guideline for implementation and local optimization, rather than a rigid, fixed protocol.

In conclusion, our model confirms the practical value of integrating diagnosis, prevention, and treatment into a structured system—achieving a balance between scientific evidence and applicability in real farm conditions.

Key conclusion: Integrated protocols provide a flexible, evidence-based framework that can be adapted to individual farm needs while maintaining high standards of herd health management.

## 7. Inferences

As a result of the reviewed established practices and protocols for monitoring, prevention, and treatment of ketosis, a summarized protocol was made for each category (Table 9) with the best possible choice in our opinion and the reason for this choice.

As a result of the literature review and the studies conducted, including a critical analysis, we systematized and summarized the already available data and reassessed the already established protocols for good veterinary practices in monitoring, prevention, and treatment of ketosis in dairy cows. A general protocol was created with a description of the methods, their advantages, disadvantages and critical notes (Table 10).

## 8. Conclusions

Based on the literature review and critical analysis, it can be confidently stated that rapid diagnosis and targeted monitoring through BHBA and F/P ratio are key for the early detection of ketosis. The implementation of prophylaxis through nutrition and oral precursors significantly reduces the incidence of the disease. The use of products such as Kexxtone (monensin) is the most effective long-term prophylactic agent in high-risk animals. The introduction of a personalized treatment protocol according to the type of ketosis increases the therapeutic success, and the training of personnel and the structured protocol (incl. data management, schedules, and monitoring) guarantee sustainable control of ketosis on farms.

## Figures and Tables

**Table 1:** Criteria for inclusion of literature in this review.

Criterion	Description
Scientific validity	Only peer-reviewed publications in journals with an impact factor/impact rank.
Current affairs	Mostly publications from the last 10–15 years, with a focus on 2012–2024.
Relevance	They relate directly to ketosis, diagnostics, therapy or metabolism.
Authority	Authors and publications with proven contributions in the field of veterinary and animal science.
Source type	Reviews, clinical trials, official guidelines.

**Table 2:** PRISMA-ScR Flow Diagram showing the selection process of studies included in the review.

Stage	Description	Number of Records
Identification	Records identified through database searching (ScienceDirect, PubMed, SpringerLink, Google Scholar)	185
	Additional records identified through other sources (conference proceedings, veterinary websites, expert suggestions)	25
Screening	Records after duplicates removed	200
	Records screened (titles and abstracts)	200
	Records excluded (irrelevant topic, non-dairy cattle, non-ketosis)	120
Eligibility	Full-text articles assessed for eligibility	80
	Full-text articles excluded, with reasons (unrelated study design, incomplete data)	40
Included	Studies included in qualitative synthesis	40
	Studies included in quantitative synthesis (if applicable)	0

Legend: PRISMA-ScR = Preferred Reporting Items for Systematic Reviews extension for Scoping Reviews. Numbers indicate the number of records at each stage.

**Table 3:** Concentrations of β-hydroxybutyrate (BHBA) (in µmol/L) in blood and their relationship to the health of dairy cattle.

BHBA Concentration (µmol/L)	Interpretation	Animal Condition	Source
<1000	Normal	No ketosis	[11,12]
1000–1200	Border zone	Potential onset of ketosis	
≥1200	Subclinical ketosis	No visible clinical signs	
≥1400–1600	Established subclinical ketosis	High risk of productive losses	
≥3000	Clinical ketosis	Signs: decreased appetite, Body Condition Score (BCS)	

**Table 4:** Comparative characteristics of ketosis types according to their origin.

Indicator	Type I Ketosis	Type II Ketosis	Silage Ketosis (Butyric)
Time of appearance	3–6 weeks after calving	1–2 weeks after calving	At any time when receiving fermented silage
Etiology	Negative energy balance (high milk yield, energy needs)	Insulin resistance, pre-calving obesity	High levels of butyrate in silage
Main reason	Lipolysis → ↑ NEFA → hepatic ketogenesis	Hepatic steatosis (fatty liver)	Increased absorption of ketone bodies from the gastrointestinal tract
Typical animal profile	A cow losing weight, but with a good appetite	Fat cow, with reduced appetite	A normal-looking cow that consumed bad silage
BHBA in the blood	≥1.2–3.0 mmol/L	≥1.2–3.0 mmol/L	Moderately elevated—from intake, not synthesis
Blood glucose	low	very high	Normal to slightly
NEFA (non-esterified fatty acids)	high	Strong	They do not increase significantly
Liver changes	Ketogenesis	Lipid infiltration (fatty liver)	Usually without steatosis
Propylene glycol response	Good	Bad	Average to good

Lipolysis → ↑ NEFA → hepatic ketogenesis: The first arrow (“→”) indicates a cause-and-effect relationship: lipolysis leads to an increase in NEFA. The second arrow (“→”) indicates the next step in the metabolic pathway: the elevated NEFA stimulate or promote ketogenesis in the liver.

**Table 5:** Preventive strategies for ketosis control in dairy cows.

Strategy/Focus Area	Implementation/Practical Recommendations	Expected Outcome/Notes
Body condition score (BCS) management	Maintain BCS 3.0–3.5 at calving; avoid overconditioning during dry period; monitor weekly prepartum	Reduces risk of type II ketosis and fatty liver; promotes optimal metabolic adaptation
Transition diet management	Provide consistent and balanced pre- and postpartum rations; increase dietary propionate precursors; avoid abrupt diet changes	Ensures smoother transition and stabilizes energy balance; reduces NEFA surge
Feed intake and housing	Provide ≥75 cm feeding space per cow; ensure feed availability 24 h/day; minimize overcrowding and heat stress	Supports higher DMI and energy intake during early lactation
Propylene glycol (PG) and propionate salts	Administer PG 250–350 g/day orally or via TMR for 3–5 days post-calving in high-risk cows; use Ca-propionate 50–100 g/day in TMR	Effective prevention of SCK; improves hepatic glucose output
Glycerin supplementation	Include 200–400 g/day in TMR or as oral drench during early lactation	Safe energy source; improves glucose availability; supports appetite
Controlled-release monensin capsules (CRC)	Single intraruminal bolus 3–4 weeks before calving, only in permitted regions	Decreases incidence of hyperketonemia; use under veterinary supervision
Mineral and vitamin support	Ensure adequate supply of cobalt, niacin, and vitamin B complex in close-up diets	Supports rumen metabolism and hepatic function
Herd-level metabolic monitoring	Regular milk F/P ratio monitoring and periodic BHBA/NEFA testing	Early identification of at-risk cows; enables timely intervention

**Table 6:** Diagnostic methods for ketosis in dairy cows.

Method (Matrix)	Diagnostic Threshold/Key Indicator	Reliability and Limitations	Recommended Use/Application
Blood BHBA	≥1.2 mmol/L (SCK threshold); ≥3.0 mmol/L (clinical ketosis)	High—gold standard for confirmation; portable meters validated in field studies	Individual cow diagnosis; confirmation of positive herd-screening results
Milk BHBA	Correlates with blood BHBA; threshold ≈ 0.10–0.15 mmol/L (varies by method)	Moderate—affected by milk yield and stage of lactation	Useful for herd-level screening where milk tests (Ketotest, FTIR) are available
Milk fat-to-protein ratio (F/P)	F/P > 1.4 indicates increased risk; >1.6–1.8 suggests SCK or energy deficit	Moderate—influenced by diet composition and stage of lactation	Suitable for herd monitoring through automated milk recording systems
Urine ketone strips (acetoacetate)	Colorimetric change indicates the presence of ketones	Low–moderate—subjective and less sensitive for early SCK	Rapid field test for clinical cases; adjunctive method
Blood NEFA	>0.4 mmol/L (prepartum risk threshold)	High—predictive for postpartum ketosis and displaced abomasum	Preventive screening in prepartum cows (−7 to 0 days before calving)
Milk citrate	<8.5 mmol/L associated with energy deficit	Low—influenced by multiple factors	Supportive indicator for metabolic monitoring at the herd level

**Table 7:** Therapeutic options for ketosis treatment in dairy cows.

Therapeutic Agent/Approach	Dosage and Route of Administration	Mechanism of Action/Expected Effect	Comments/Recommendations
Propylene glycol (PG)	250–500 g per day orally for 3–5 consecutive days	Gluconeogenic precursor; increases plasma glucose and decreases BHBA	First-line therapy for SCK and supportive treatment for clinical ketosis
Intravenous glucose (dextrose 50%)	500 mL IV once daily for 1–3 days	Rapid elevation of blood glucose; transient reduction in ketone bodies	Short-term effect; combine with PG for sustained response
Glucocorticoids (dexamethasone, isoflupredone acetate)	Dexamethasone 10–20 mg IM once; Isoflupredone 5–10 mg IM once	Induces gluconeogenesis and appetite stimulation	Use with caution; may cause immunosuppression or induce calving if overdosed
Glycerin (glycerol)	200–400 g/day orally or in TMR for 5–7 days	Alternative glucogenic substrate; stimulates insulin secretion	Safe and well tolerated; slower effect compared to PG
Calcium propionate	50–100 g/day orally or in TMR for 5 days	Provides propionate for gluconeogenesis and a calcium source	Effective as adjunct preventive and supportive treatment
Insulin (regular insulin)	0.25–0.5 IU/kg SC or IM, combined with glucose infusion	Enhances glucose utilization and decreases lipolysis	Used in severe clinical ketosis; requires veterinary supervision
Monensin (rumen modulator)	300 mg/day via controlled-release capsule (CRC)	Alters rumen fermentation to increase propionate production	Preventive use; not recommended as a stand-alone treatment
B-complex vitamins (B12, niacin)	As per label dosage (commonly 10–20 mg niacin/day)	Improves hepatic metabolism and reduces fatty infiltration	Supportive therapy during the recovery phase

**Table 8:** Integrated GVP protocol for monitoring, prevention, and treatment of ketosis in dairy cows.

Stage/Focus	Recommended Actions (Good Veterinary Practice Protocol)	Key Parameters to Monitor	Responsible Personnel/Frequency
Prepartum (−3 to 0 weeks before calving)	-Evaluate BCS (target 3.0–3.5). -Introduce a transition diet with balanced energy density. -Administer controlled-release monensin capsule (where approved). -Monitor NEFA levels (>0.4 mmol/L = risk).	BCS, NEFA, diet composition	Veterinarian/farm nutritionist; weekly
Early lactation (0–21 days postpartum)	-Observe DMI and feeding behavior daily. -Screen milk F/P ratio (>1.4 = risk). -Test BHBA in blood (≥1.2 mmol/L = SCK; ≥3.0 mmol/L = CK). -Administer propylene glycol 250–350 g/day for 3–5 days in high-risk cows.	BHBA, milk F/P ratio, DMI	Herd manager/veterinarian; 2–3 times weekly
Clinical case management	-Confirm diagnosis with blood BHBA or NEFA. -Administer IV glucose (500 mL 50%) + PG orally for 3–5 days. -Add dexamethasone (10–20 mg IM) in severe cases. - Support with vitamin B complex.	BHBA response, clinical signs, appetite	Veterinarian; daily until recovery
Prevention and nutritional follow-up	-Adjust ration to ensure adequate propionate precursors (PG, glycerin, Ca-propionate). -Monitor milk production and feed intake trends. -Continue PG supplementation for at-risk multiparous cows.	Milk yield, F/P ratio, body weight	Nutritionist/herd manager; weekly
Herd-level monitoring and evaluation	-Record SCK prevalence and treatment outcomes. -Review metabolic test data quarterly. -Update herd protocols annually based on results and new evidence.	SCK rate, treatment success, economic losses	Veterinarian/herd consultant; quarterly

**Table 9:** Summary protocol for method selection and rationale.

Category	Best Choice	Reason
Monitoring	BHBA in blood + F/P in milk	Highest sensitivity and practicality
Prevention (small herds)	Propylene glycol + BCS control	Economical and effective approach
Prevention (large herds)	Kexxtone + feed-grade glycerin	Long-term protection, lower labor resources
Treatment (mild ketosis)	Propylene glycol	Proven effect of early intervention
Treatment (severe ketosis)	IV glucose + insulin (veterinarian)	Rapid recovery in clinical form

**Table 10:** Summary protocol for good veterinary practices in ketosis.

Stage/Component	Method/Action	Purpose/Benefits	Critical Notes
Risk assessment	-BCS (3.0–3.5)- NEFA before calving (<0.3 mmol/L)- F/P <1.4	Ketosis risk prediction	NEFA > 0.4 = risk; F/P > 1.5 = subclinical ketosis
Monitoring (incremental)	-BHBA in blood (≥1.2 mmol/L) -Ketone bodies in milk (ketotest) -F/P analysis -Observation: appetite, milkiness	Timely detection of subclinical and clinical ketosis	BHBA is the gold standard; F/P is easy but indirect
Prevention—nutrition	-Limited ration in dry period (75–80%) -Addition of straw -Gradual addition of concentrate before calving	Reducing NEB, preventing ketosis type I and II	Avoiding silage with butyric acid
Prevention—supplements	-Propylene glycol: 250–350 g/day -Calcium propionate: 450 g/day -Kexxtone: 3–4 weeks before calving (bolus)	Supporting gluconeogenesis, reducing βOHB	Kexxtone is effective but requires a veterinarian and precise application
Subclinical ketosis	-Propylene glycol (300 mL, 3–5 days) -Glycerin/propionates -Retest	Preventing progression to clinical ketosis	Without glucocorticoids
Clinical ketosis	-IV glucose (500 mL) -B-vitamins -Propylene glycol -Glucocorticoids (type I only)	Rapid correction of hypoglycemia, improvement of condition	Glucocorticoids are contraindicated in type II ketosis (with steatosis)
Tools and tests	-Precision Xtra -Ketotest -Tank milk analysis (F/P) -NEFA analysis	Precise monitoring, tracking of metabolic status	Portable devices allow field testing
Performance evaluation	-Increased appetite -Reduced βOHB values -Increased milk production -Documented treatment	Objective monitoring of response to treatment	Keeping individual cards and records
Staff training	-Recognizing symptoms -Administering a bolus -Working with a ketometer	Error reduction, early intervention	Key to successful prevention in large herds

## Data Availability

No new data were created or analyzed in this study.
